# Triptolide enhances carboplatin-induced apoptosis by inhibiting nucleotide excision repair (NER) activity in melanoma

**DOI:** 10.3389/fphar.2023.1157433

**Published:** 2023-06-01

**Authors:** Geng Wang, Hongmin Guo, Yan Ren, Weiyi Chen, Yixuan Wang, Jianing Li, Hua Liu, Jingjun Xing, Yanru Zhang, Na Li

**Affiliations:** ^1^ Health Science Center, Ningbo University, Ningbo, China; ^2^ People’s Hospital of Changshou Chongqing, Chongqing, China

**Keywords:** melanoma, triptolide, carboplatin, nucleotide excision repair (NER), DNA damage

## Abstract

**Introduction:** Carboplatin (CBP) is a DNA damaging drug used to treat various cancers, including advanced melanoma. Yet we still face low response rates and short survival due to resistance. Triptolide (TPL) is considered to have multifunctional antitumor effects and has been confirmed to enhance the cytotoxic effects of chemotherapeutic drugs. Herein, we aimed to investigate the knowledge about the effects and mechanisms for the combined application of TPL and CBP against melanoma.

**Methods:** Melanoma cell lines and xenograft mouse model were used to uncover the antitumor effects and the underlying molecular mechanisms of the alone or combined treatment of TPL and CBP in melanoma. Cell viability, migration, invasion, apoptosis, and DNA damage were detected by conventional methods. The rate-limiting proteins of the NER pathway were quantitated using PCR and Western blot. Fluorescent reporter plasmids were used to test the NER repair capacity.

**Results:** Our results showed that the presence of TPL in CBP treatment could selectively inhibit NER pathway activity, and TPL exerts a synergistic effect with CBP to inhibit viability, migration, invasion, and induce apoptosis of A375 and B16 cells. Moreover, combined treatment with TPL and CBP significantly inhibited tumor progression in nude mice by suppressing cell proliferation and inducing apoptosis.

**Discussion:** This study reveals the NER inhibitor TPL which has great potential in treating melanoma, either alone or in combination with CBP.

## 1 Introduction

Melanoma is a skin tumor with a rising incidence based on the number of cases and deaths from 2016 to 2020. The annual number of new cases and deaths per 100,000 men and women is 21.0 and 2.1, respectively (http://seer.cancer.gov/statfacts/html/melan.html). Until recently, alkylating agents were still the basic treatment modality in metastatic melanoma, yet the response rates were less than 10%–15% (Middleton et al., 2000; Patel et al., 2011). Although emerging immunotherapies and targeted therapies have achieved some success in recent years, chemotherapy remains an important treatment option for metastatic melanoma worldwide (Villaruz et al., 2015). Therefore, new therapeutic anticancer drugs and more effective therapeutic strategies are necessary for melanoma therapy.

**FIGURE 1:**
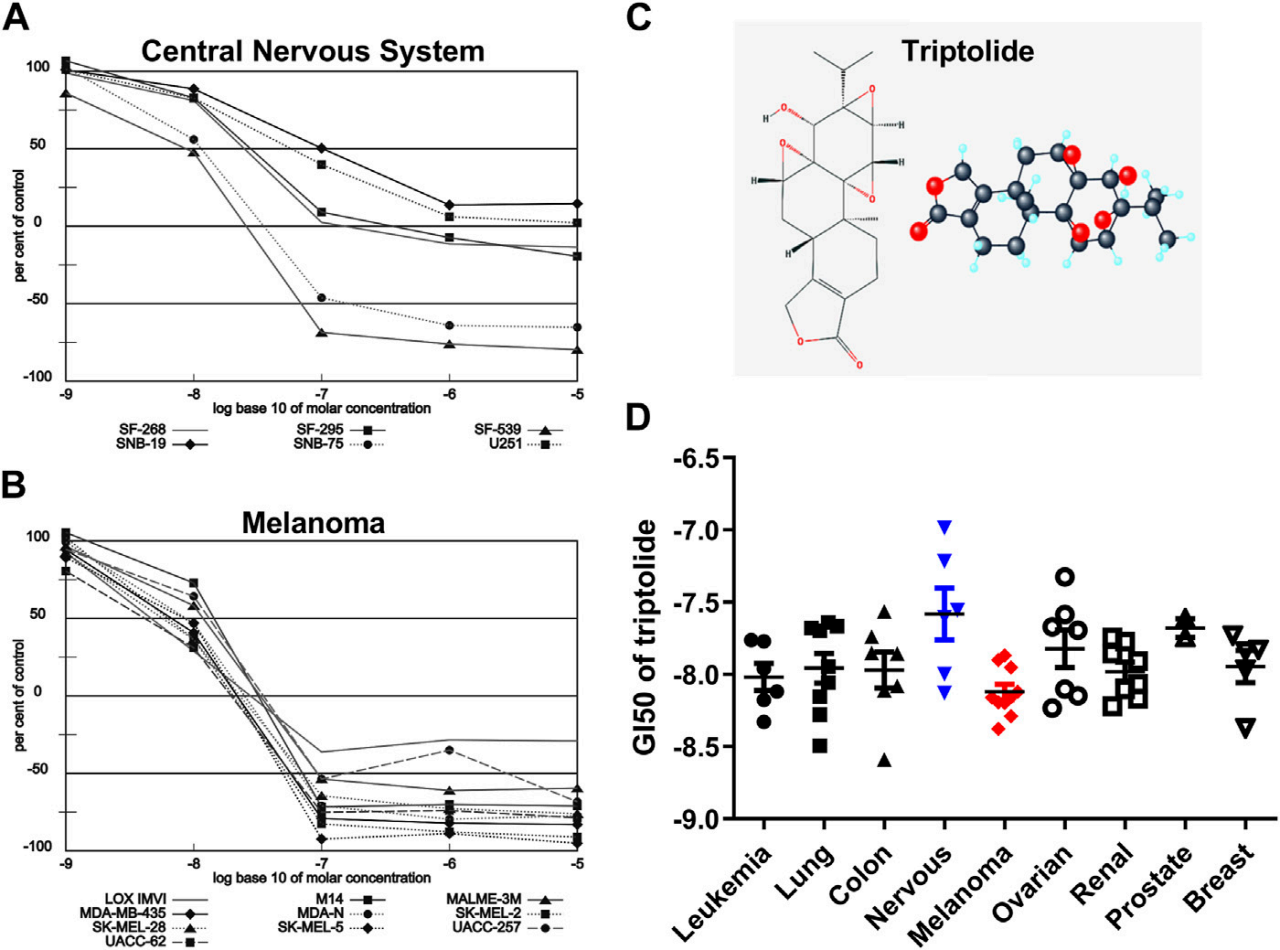
Melanoma is more sensitive to TPL than the other tissues in the NCI-60 database. Dose-response curves of **(A)** Melanoma and **(B)** Central Nervous System cell lines to TPL (Compound 163062). **(C)** 2D and 3D structures of TPL. **(D)** The difference in Growth inhibition 50 (GI_50_) of TPL between different tissue-derived cell lines (one-way *ANOVA* with *post hoc* test).

Triptolide (TPL) is one of the most biologically active extracts from the Thunder God Vine, *Tripterygium wilfordii*, which has already been used to treat inflammation and autoimmune disease in traditional Chinese medicine for centuries (Liu, 2011). The 2D and 3D structures of TPL are shown in Figure 1C. TPL has also been demonstrated to possess anti-fertility, anti-cystogenesis, anti-angiogenesis, and anticancer activities, which can inhibit the growth and the development of various types of solid tumors, including melanoma (Lue et al., 1998; Liu, 2011; Tao et al., 2012; Wang et al., 2017; Zhao et al., 2020). The NCI-60 database showed that TPL exhibited notable anticancer activity at nanomolar concentrations like the paclitaxel and doxorubicin (https://dtp.cancer.gov/discovery_development/nci-60). The cell lines from melanoma were more sensitive to TPL than the other cells from eight different human tissues, especially from the central nervous system (Figure 1). Furthermore, TPL is considered to have multifunctional antitumor effects, such as activation of apoptosis, interactions with RNA polymerase II complex to induce a global suppression of RNA expression, anti-angiogenesis, and targeting of cancer stem cells (Hou et al., 2018). Therefore, TPL might be a more effective chemotherapy drug for the treatment of melanoma.

Carboplatin (CBP) is a platinum drug that exhibits its cytotoxic effects by forming intra- and inter-strand cross-linked DNA adducts, which eventually distorts the DNA and causes DNA damage. The distorted DNA can be identified and repaired mainly through the nucleotide excision repair (NER) pathway, which is one of the reasons for the failure of platinum-based therapies (Chaney and Vaisman, 1999; Siddik, 2003; Reed, 2005; McNeil and Melton, 2012; Modi et al., 2016). NER contains a couple of crucial proteins for DNA damage recognition and excision, plus a helicase (Salehan and Morse, 2013). The elevated level of NER factors has been indicated or proved to be implicated in poor response and resistance to platinum-based treatments in many types of cancers (Aloyz et al., 2002; McNeil and Melton, 2012; Salehan and Morse, 2013; Modi, et al., 2016). It is widely accepted that TPL can bind to a subunit of TFIIH basal transcription factor XPB, resulting in the repression of RNA polymerase II-mediated transcription. In fact, XPB is not only related to transcription but also inseparable from the processes of NER (Aloyz, et al., 2002; Titov et al., 2011; Salehan and Morse, 2013; He et al., 2015). TPL has also been confirmed to enhance the cytotoxic effects of other chemotherapeutic drugs in various cancers (Li et al., 2012; Zhu et al., 2012; Chen et al., 2014; Ho et al., 2015). However, knowledge about the relevance of both XPB and other NER genes for the combined application of TPL and CBP in melanoma still needs to be implemented.

In this work, we have detected the effects of the alone or combined treatment of TPL and CBP on melanoma cells *in vitro* and *in vivo*, and examined whether TPL affected CBP-induced DNA damage repair efficiency by affecting the NER pathway. We hypothesized that TPL might be used as a drug-sensitizer to enhance the effect of tumor cells on DNA damaging drug therapy by selectively inhibiting the NER activity and increasing the extent of apoptosis.

## 2 Materials and methods

### 2.1 Cell culture, plasmids, and drugs

A375 and B16 melanoma cell lines were purchased from National Collection of Authenticated Cell Cultures. Both cells were maintained in DMEM medium (Biological Industries, BI, Kibbutz Beit-Haemek, Israel), as we mentioned before (Li et al., 2023).

The pCMV-C-DsRed (D2624) and pCMV-C-EGFP (D2626) plasmids were purchased from Beyotime (China), which carried a CMV promoter-driving Discosoma sp. red fluorescent protein gene and a green fluorescence protein gene, respectively. Both the two plasmids were amplified in *E. coli* DH5α strain and purified using a Plasmid Maxi Preparation Kit (Beyotime, China).

Triptolide (Biopurify Phytochemical Ltd., Chengdu, China) was dissolved in dimethyl sulfoxide (DMSO) (10 mM in stock). The final concentration of DMSO was controlled at <0.1% in all experiments. Carboplatin (Selleck Chemicals, Shanghai, China) was dissolved in water as a 10 mM solution and used freshly.

### 2.2 Antibodies

For the apoptosis analysis of the cells after treatments, the primary antibodies used were as follows: Cleaved caspase-3 (Asp175, #9661), γH2A.X (Phospho-Histone H2A.X, Ser139, #9718) and p53 (#2524) antibodies were purchased from Cell Signaling Technology (CST, United States); caspase-3 (AF1213), PARP (AF1657), cleaved PARP (AF1567), Ki67 (AF1738), and β actin (AF5003) antibodies were obtained from Beyotime.

To explore the activation of the NER signal pathway, the primary antibodies used were as follows: ERCC1 (#12345), ERCC4/XPF (#13465), and XPC (#12701) antibodies were obtained from CST; ERCC3/XPB (sc-271500) antibody was purchased from Santa Cruz Biotechnology (United States); ERCC2/XPD (10818-1-AP), ERCC5/XPG (67055-1-Ig), and XPA (16462-1-AP) antibodies were purchased from Proteintech (WUHAN SANYING, China). Protein expression levels were expressed relative to β actin by calculating band intensity values. Anti-mouse IgG and anti-rabbit IgG were used as HRP-conjugated secondary antibodies.

### 2.3 Cell viability assay and combination effect analysis

MTT method was used to determine cell viability, as we mentioned before (Wang, et al., 2017). In brief, A375 and B16 cells were seeded into 96-well plates one night before, and then the indicated concentrations of drugs were used to treat the cells for 24, 48 and 72 h. Finally, MTT was used to detect the amount of remaining live cells. All the experiments were performed in triplicate and independently repeated three times.

Isobologram analysis method (Steel and Peckham, 1979) was used to analyze the combined effect of TPL and CBP on the cancer cells of melanoma. The dose-response curves of the two drugs, alone or in combination, were depicted by the MTT method. When TPL and CBP were used in combination, a constant ratio of 1:2000 was maintained throughout. The isobologram of the two drugs was automatically constructed using CompuSyn software (ComboSyn, Inc., United States), which was designed by Chou-Talalay. The combination index (CI)-isobologram equation could be used to quantify the drug combination effects, where CI < 1, = 1, and >1 indicated synergism, additive effect and antagonism, respectively.

### 2.4 Colony formation assay

Cells were counted and seeded into six-well plates overnight at proper densities, and then treated with indicated drug dosages for 5–7 days to form colonies. The cells were fixed in 4% paraformaldehyde for 30 min and stained with 0.25% crystal violet for another 30 min at room temperature. The plates were washed with distilled water, allowed to dry overnight, and finally scanned with a scanner. The colonies were quantified by ImageJ software.

### 2.5 Apoptosis assay

Cells were seeded into six-well plates overnight and then treated with indicated drug dosages for 48 h. Both the floating and adherent cells were used for the apoptosis assay. Annexin V-FITC/7-AAD apoptosis detection kit (Sino Biological Inc., China) and flow cytometry analysis were used to determine the ratio of the apoptotic and necrotic cells.

### 2.6 Comet assay for DNA damage

A375 cells were seeded in six-well plates overnight at the density of 5×10^5^ cells/well, and then incubated with vehicle (DMSO), 30 nM TPL, 50 μM CBP, and 30 nM TPL +50 μM CBP for 48 h. DNA damage in treated cells was measured using the comet assay as described previously (Olive and Banath, 2006). Briefly, normal agarose was heated and spread on slides until it solidified. Then the slides were coated with a mixture of the treated cells and 1% low gelling temperature agarose. After mixture solidification, the slides were immersed in lysis solution (pH 13) for 2 h and subjected to electrophoresis. DNA and its fragments were stained with ethidium bromide in PBS and photographed with a fluorescence microscope. DNA breaks in each individual nucleus were assessed by calculating the relative length and intensity of DNA tails to heads using the CSAP comet analysis software project (V1.2.2). In this assay, the Olive tail moment (TM) was calculated as a parameter for assessing the extent of DNA breaks. The TM value was the product of the tail length and the fraction of total DNA in the tail.

### 2.7 Caspase-3 and caspase-9 activity assay

Caspase activities were measured using colorimetric activity assay kits (Beyotime, C1116 and C1157, China). The principle of this assay is that the chromogenic substrates, Ac-DEVD-*p*NA and Ac-LEHD-*p*NA, can be cleaved by caspase-3 and caspase-9, respectively. Briefly, the treated cells were lysed on ice and centrifuged to obtain the supernatant with proteins. Then the supernatant was incubated with caspase substrate solution for 2 h at 37°C. The absorbance values were measured by a Thermo Fisher Multiskan FC microplate reader at 405 nm.

### 2.8 Animals and xenograft studies

Female BALB/c nude mice (4–6 weeks old) were purchased from Beijing Vital River Laboratory Animal Technology and housed in SPF class small rodent center. All procedures were performed in accordance with the guidelines of the Research Ethics Committee of Ningbo University (Permit No. 10498).

The A375 cells from liquid nitrogen were thawed and resuspended in 100-mm cell culture dishes. The inoculation density should be about 70% of the culture dish, which is covered on the third day. The cells were then collected and resuspended in serum-free culture medium. The cells should be filtered with a 40 μm filter to inoculate mice as soon as possible. A375 cells were resuspended in 40% Matrigel (BD Biosciences) on ice, and then injected subcutaneously into the right axilla of the nude mice. Tumors were measured in two dimensions using an electronic vernier caliper every 2 days. Tumor volume (TV) was calculated using the formula as (length × width^2^)/2. Upon harvesting, tumors were fixed in formalin overnight and then in 70% ethanol for histopathology analysis.

### 2.9 Drug administration

The day of the TV reaching approximately 100-mm^3^ was counted as day 0. Then the tumor-bearing mice were randomly divided into four groups and injected intravenously with vehicle 0.9% saline, TPL, CBP, and TPL + CBP, respectively, with 6 mice in each group. The dosages of TPL and CBP in all groups were 0.15 mg/kg/day and 20 mg/kg/3 days, respectively. The volume of each injection was 200 μL.

### 2.10 NER repair capacity analysis

Fluorescent reporter plasmids were used to test the NER repair capacity, and similar experimental procedures such as the host cell reactivation (HCR) assay had been described before (Nagel et al., 2014; Ciminera et al., 2021). In this experiment, a plasmid containing a fluorescent reporter gene was damaged by CBP and then transfected into cells to be assessed for repair. Under normal conditions, cells can repair most of the fluorescent reporter genes in CBP-damaged plasmid DNA through the NER pathway. Therefore, fluorescence intensity can be used to indicate the degree of repair in damaged plasmid DNA.

DNA damage in plasmid DNA was generated by incubating the pCMV-C-EGFP plasmid DNA with 50 μM CBP in 10 mM Tris (pH8.5) for 6 h 37°C. To remove free CBP, the CBP-damaged EGFP plasmid DNA was precipitated by 70% ethanol, dissolved in 10 mM Tris (pH8.5), and then transfected into treated cells. The pCMV-C-DsRed (Discosoma sp. Red) plasmid was used as an endogenous transfection control.

For transfection, A375 cells were seeded in six-well plates overnight and then co-transfected with both CBP-damaged EGFP (2 μg) and DsRed (2 μg) plasmid DNA by Lipo8000™ Transfection Reagent (Beyotime) per the manufacturer’s instructions. Transfected cells were treated with or without TPL (30 nM) for 24 h at 37°C to monitor the changes in NER repair capacity. Fluorescent reporter expression was visualized by fluorescence microscope and analyzed by flow cytometry. The black-white images of the same view were also recorded simultaneously to visualize the cells. NER repair capacity was calculated as the percentage of fluorescent reporter expression using FlowJo. The fluorescent signal was computed using the formula of “*F* = (*N* × *MFI*)/S”. Where *S* is the total number of live cells and *N* is the number of EGFP or DsRed positive cells. *MFI* is the mean fluorescence intensity of the *N* cells. The DsRed and EGFP fluorescence signals were designated as *Fc* and *Fg*, respectively. The normalized EGFP fluorescent signal *Fn* was calculated using a formula of “*Fn = Fg/Fc*”.

### 2.11 Statistical analysis

Values are expressed as mean ± standard deviation unless otherwise noted. Data were analyzed in GraphPad Prism (v8.0.2) by unpaired Student’s *t*-test, or one-way *ANOVA* with a *post hoc* test. ^*^ *p* < 0.05, ^**^ *p* < 0.01, and ^***^ *p* < 0.001 were considered to be statistically significant. When processing some data, a log transformation was performed to generate a Gaussian distributed dataset.

## 3 Results

### 3.1 Melanoma is more sensitive to TPL than the other tissues in the NCI-60 database

The NCI-60, a panel of 60 cancer cell lines from various human tissues, has been used to screen hundreds of thousands of chemical compounds and natural products (since 1990). The NCI-60 database showed that the cell lines from melanoma were more sensitive to TPL than the other cells from eight different human tissues (Figure 1D, one-way *ANOVA*, *p* = 0.0463). However, the cell lines from the central nervous system were more resistant to TPL (Figure 1D). Dose-response curves of Melanoma (Figure 1A) and Central Nervous System (Figure 1B) cell lines to TPL showed that all melanoma cells were similarly sensitive to TPL and responded well to TPL. It can be clearly seen from the curves that when the TPL concentration is 100 nM (the value on the *X*-axis is −7), most central nervous system cell lines are more resistant relative to melanoma cell lines. Therefore, TPL may be an effective drug for the treatment of melanoma with tissue specificity and lower neurotoxicity.

### 3.2 Combined therapy with TPL and CBP synergistically inhibits viability of melanoma cells

**FIGURE 2:**
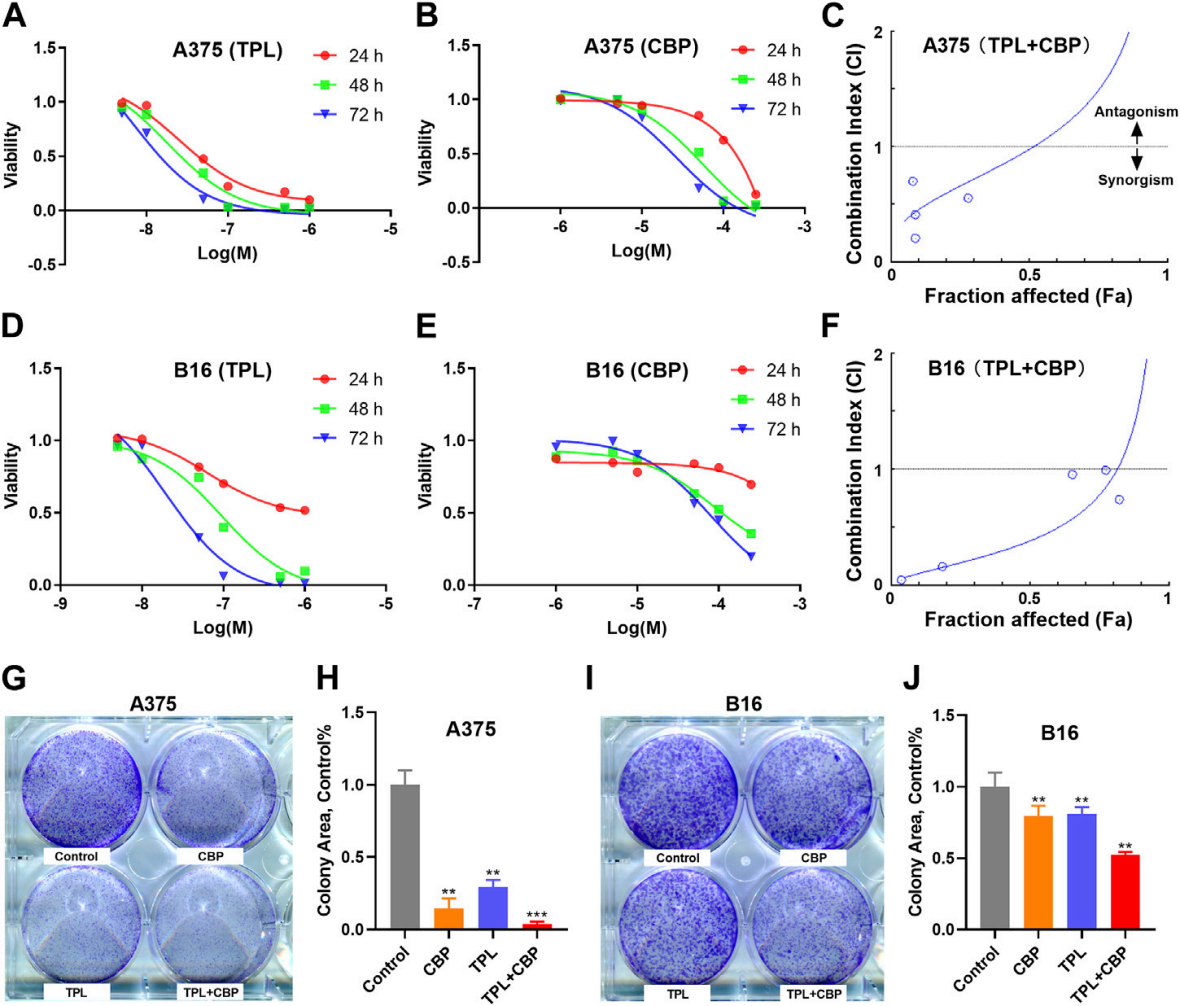
Effect of TPL and CBP on the growth of A375 and B16 cells *in vitro*. **(A,B)** A375 and **(D,E)** B16 cells were incubated with TPL (5, 10, 50, 100, 500, and 1,000 nM) or CBP (1, 5, 10, 50, 100, and 250 μM) for 24, 48, and 72 h, and the proliferation of the treated cells was determined by MTT method. **(C,F)** The effects of combined treatment of A375 and B16 cells with TPL and CBP were analyzed by Combination Index-Isobologram Equation analysis, where Combination Index (CI) < 1, = 1, and >1 indicated synergism, additive effect and antagonism, respectively. **(G,I)** Representative colony formation assays with TPL (1 nM) and CBP (2.5 μM) on A375 or B16 cells alone or in combination. **(H,J)** The statistical analysis of the colony formation assay.

The proliferation of A375 and B16 cells treated with TPL or CBP for 24, 48 and 72 h were measured by the MTT method. As seen in Figures 2A, B, D, E, the growth of A375 and B16 cells was significantly suppressed by TPL and CBP in a dose-dependent manner. TPL inhibited the proliferation of A375 and B16 cells with the estimated IC_50_ at 14.7 and 30.1 nM, respectively. CPB inhibited the proliferation of A375 and B16 cells with the IC_50_ at 43.4 and 72.9 μM, respectively. TPL enhanced the efficacy of CBP and indicated a synergistic effect in the treatment of A375 and B16 cells (Figures 2C, F). The fraction affected (Fa) *versus* combination index (CI) curves shown in Figures 2C, F demonstrate the synergistic (CI < 1) anti-proliferative effect of TPL combined with CBP.

The colony formation assay was also used to assess the synergism of TPL and CBP. We subjected A375 and B16 cells to 1 nM TPL and 2.5 μM CBP alone or in combination, respectively. The viability results showed that the combination of TPL and CBP produced a greater growth inhibition effect in melanoma cells than either drug alone (Figures 2G–J).

### 3.3 Combined therapy with TPL and CBP synergistically inhibits migration and invasion of melanoma cells

**FIGURE 3:**
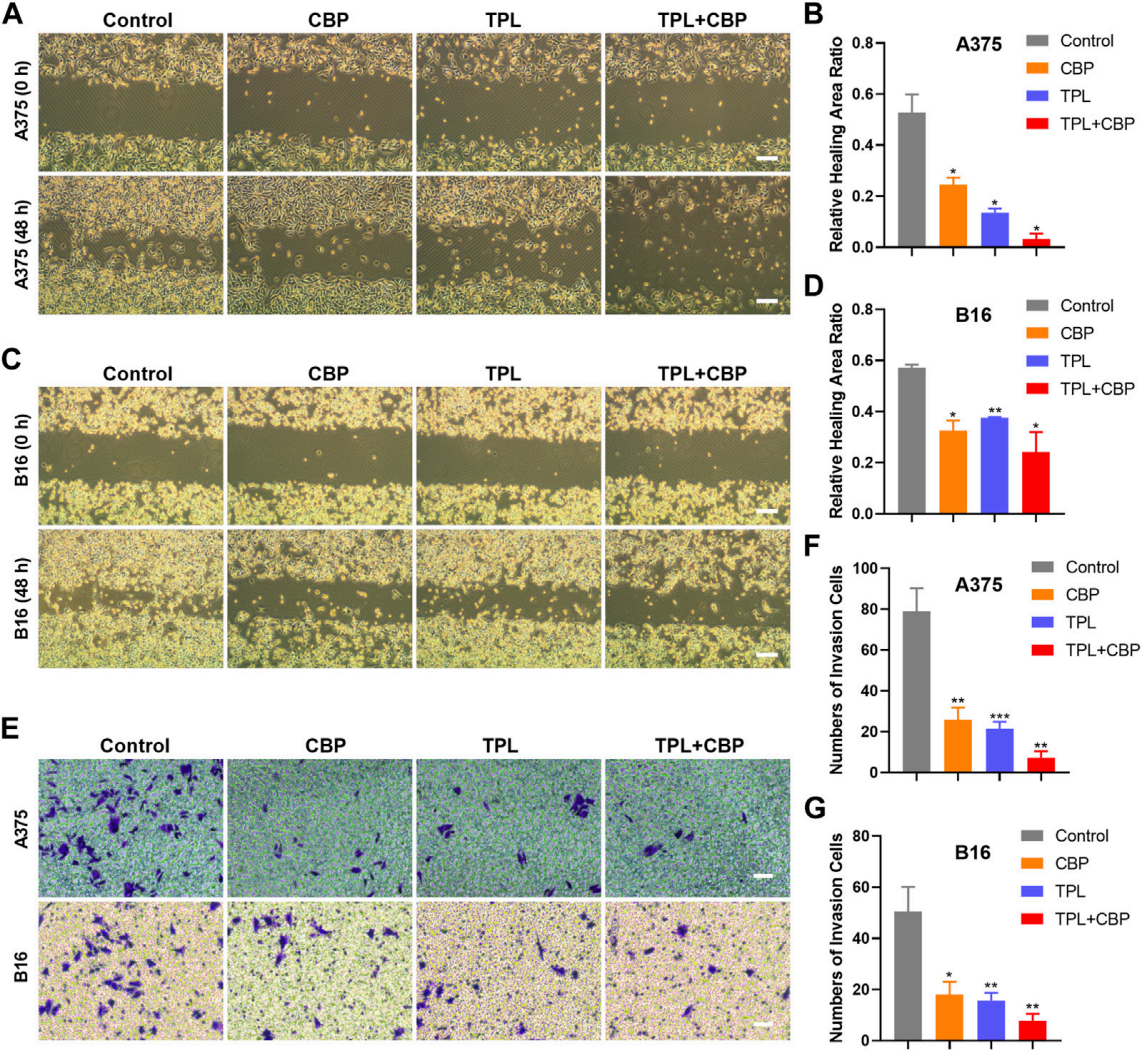
Effect of TPL and CBP on migration and invasion of A375 and B16 cells *in vitro*. The wound-healing assay and transwell assay were used to evaluate the cell migration and invasive ability of the treated melanoma cells, respectively. Representative migration images and statistical analysis of A375 **(A,B)** and B16 **(C,D)** cells treated with TPL, CBP, or a combination. Scale bar = 200 μm. Representative transwell images and statistical analysis of A375 **(E,F)** and B16 **(E,G)** cells treated with TPL, CBP, or a combination. Scale bar = 50 μm.

Given the close relationship between the migration and invasion of cancer cells and tumor progression, the effects of TPL and CBP on migration and invasion were evaluated. The analysis results of cell migration by wound healing assay showed that TPL or CBP used alone remarkably repressed A375 (Supplementary Figure S1A–D) and B16 (Supplementary Figure S1E–H) cell migration in a dose-dependent manner. When they used together, the migration ability was significantly inhibited in A375 (Figures 3A, B) and B16 (Figures 3C, D) cells.

Changes in cellular permeability and chemotactic capacity, i.e., changes in invasiveness, of treated melanoma cells were determined by transwell assay. The results indicated that the number of invasive A375 (Figures 3E, F) and B16 (Figures 3E, G) cells treated with TPL or CBP was significantly reduced compared to the control, and co-treatment with both drugs significantly inhibited the invasive ability in a synergistic manner. These results proved that TPL combined with CBP reduced the chemotactic capability and penetration efficiency of melanoma cells, thereby synergistically inhibiting tumor cell migration and invasion.

### 3.4 Combined therapy with TPL and CBP synergistically induces apoptosis

**FIGURE 4:**
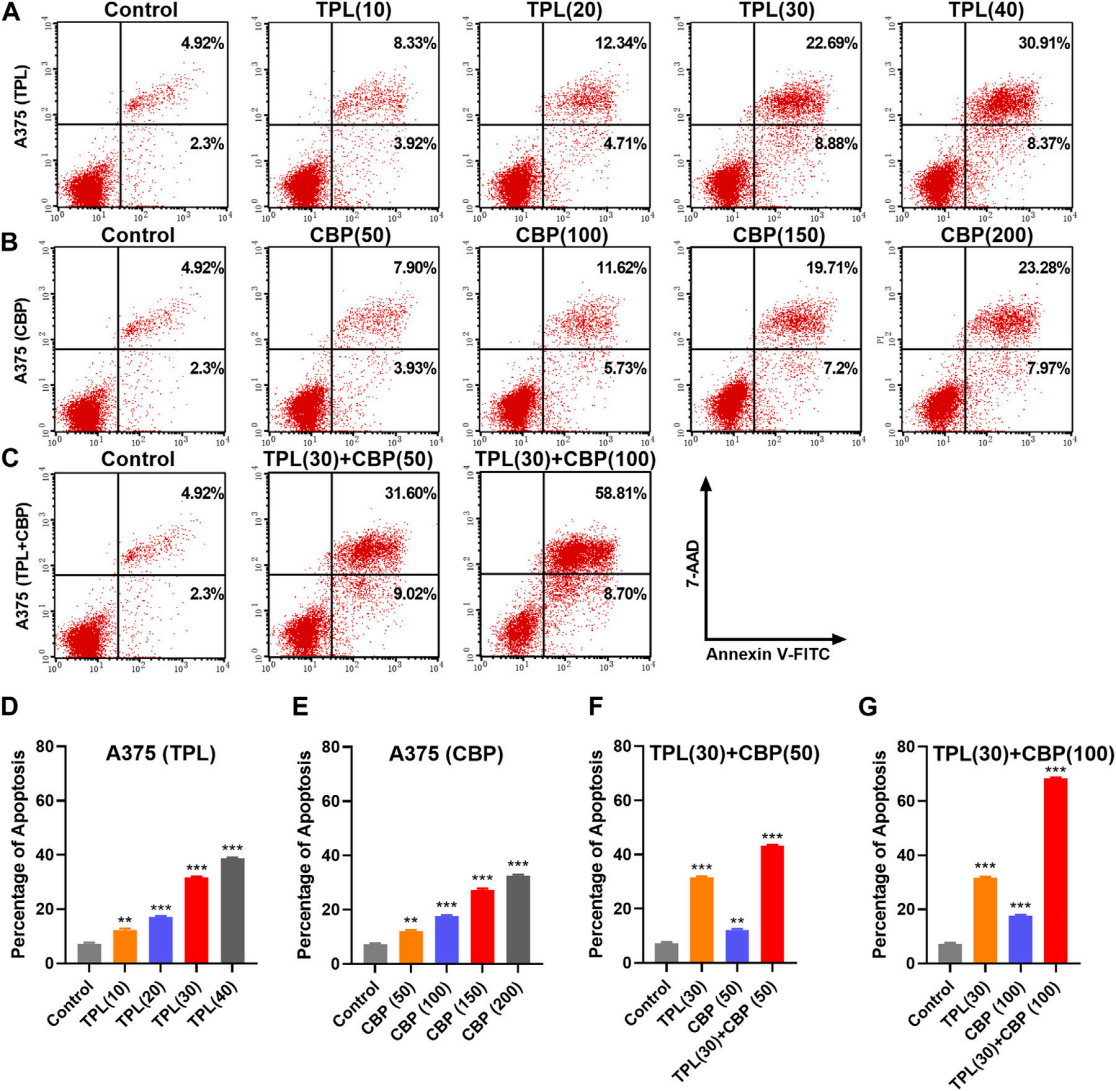
Apoptosis induced by TPL and CBP in A375 cells. Apoptosis of **(A)** TPL (0, 10, 20, 30, 40 nM), **(B)** CBP (0, 50, 100, 150, 200 μM), and **(C)** TPL (30 nM) combined with CBP (50 or 100 μM) treated A375 cells at 48 h was detected by double staining of annexin V-FITC and 7-AAD. **(D–G)** The statistical analysis of the annexin V apoptosis assay.

Platinum drugs mainly play their role by inducing cell apoptosis (Siddik, 2003). The apoptotic rates in TPL and CBP treated A375 cells were evaluated using the annexin V apoptosis assay and were determined by flow cytometry. Results showed that the percentage of apoptotic cells induced by TPL (Figures 4A, D) and CBP (Figures 4B, E) increased in a dose-dependent manner. Combined therapy with TPL and CBP remarkably increased the apoptotic rates of A375 cells (Figures 4C, F, G), indicating that the combined therapy synergistically induced more cell death than the use of drugs individually.

### 3.5 Regulatory mechanisms of TPL and CBP induced apoptosis in melanoma cells

**FIGURE 5:**
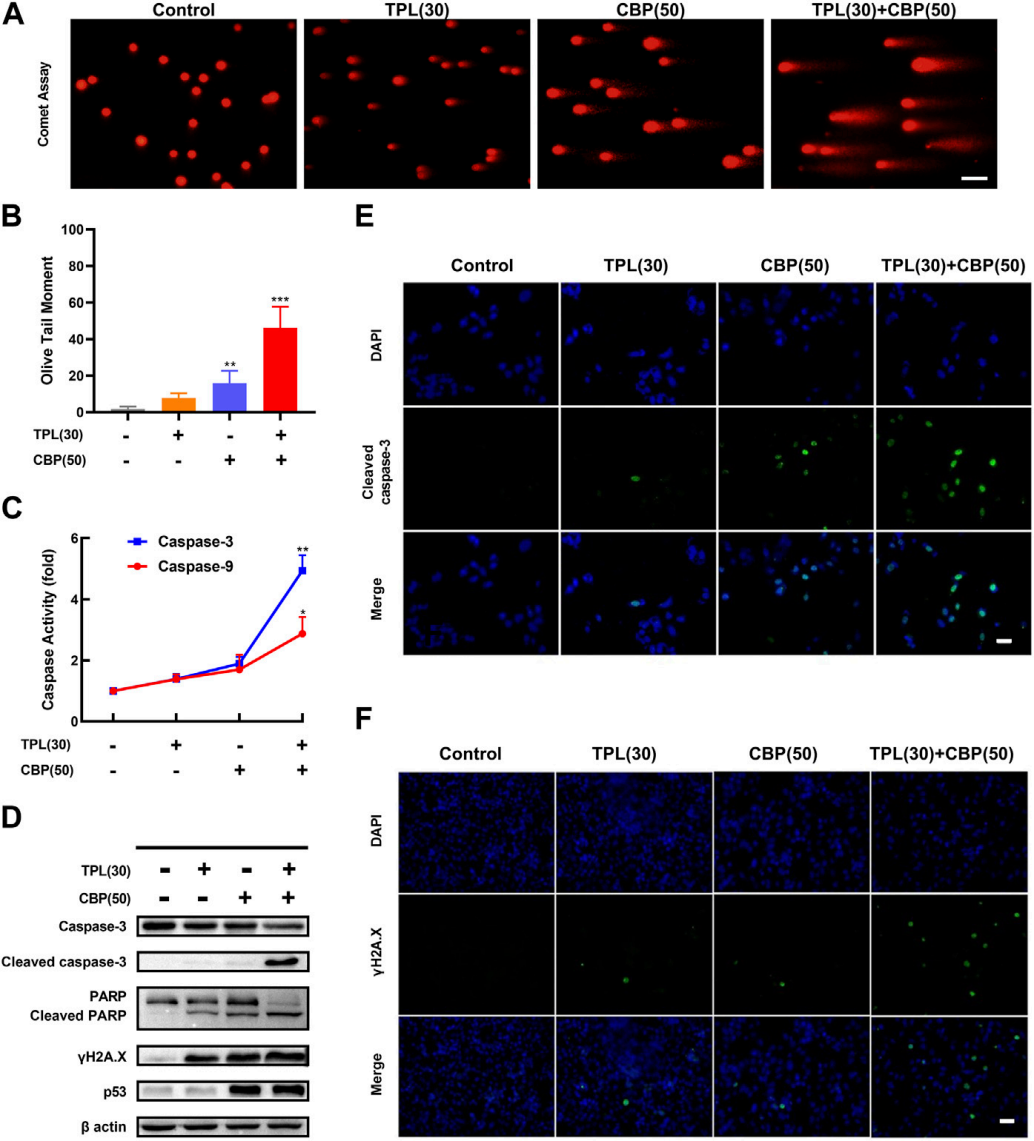
DNA damage and caspase activation induced by TPL and CBP in A375 cells. **(A)** The extent of chromatin fragmentation induced by TPL and CBP in A375 cells. **(B)** Olive tail moment method was used to analyze the DNA damage status. **(C)** Activity of caspase-3 and -9 after 24 h treatment with 30 nM TPL and 50 μM CBP in A375 cells, data are expressed as fold change relative to untreated control. **(D)** Protein expression of caspase-3, cleaved caspase-3, PARP, cleaved PARP, γH2A.X and p53 in A375 cells was determined by Western blot analysis at 48 h after treatment with 30 nM TPL and 50 μM CBP alone or in combination. **(E,F)** Immunostaining of cleaved caspase-3 and γH2A.X in TPL and CBP treated A375 cells after 48 h treatment by immunofluorescence microscopy. Scale bar = 50 μm.

Since platinum drugs like CBP can induce double-strand breaks in DNA due to the formation of platinum adducts. DNA damage was assayed in TPL and CBP treated A375 cells by determining the extent of chromatin fragmentation using a comet assay. DNA fragments were clearly detectable in TPL and CBP co-treated A375 cells. However, no significant DNA fragments were observed in TPL (30 nM) treated cells (Figures 5A, B).

Since γH2A.X (Phospho S139) is a sensitive biomarker of DNA damage and repair (Mah et al., 2010; Ivashkevich et al., 2012), and platinum drugs such as CBP are known to induce DNA damage, we evaluated the protein expression of phosphorylated H2A.X by Western blot and immunofluorescence assays following TPL, CBP, and the combination treatment. Our results showed that the expression of phosphorylated H2A.X increased when the A375 cells were treated with 30 nM TPL or 50 μM CBP after 48 h. Combined therapy with TPL and CBP significantly increased γH2A.X expression compared to either drug alone (Figures 5D, F). Based on these results, we concluded that treatment with CBP did cause DNA damage; meanwhile, this damage was further exacerbated by co-treatment with TPL and CBP.

To investigate the mechanisms of apoptosis in TPL and CBP treated A375 cells, the activation of caspases was determined by caspase activity assay; the expression of apoptotic proteins was determined by Western blot and immunofluorescence. The expression of cleaved caspase-3 and cleaved PARP was used as molecular markers of apoptosis. In cells co-treated with TPL and CBP for 48 h significantly enhanced the cleavage of caspase-3 and the downstream PARP compared to either drug alone (Figures 5D, E). Tumor suppressors such as p53 engage apoptotic pathways through activating canonical DNA damage sensors, in response to DNA-damaging agents, like platinum agents and UV, X or Y irradiation (Lowe, 1995). Our results showed that the co-treatment group had an increased level of p53 (Figure 5D), whereas this dose of TPL did not cause a significant increase in p53 expression.

Our results showed that the caspase-3 and -9 activities were raised when the cells were treated with 30 nM TPL or 50 μM CBP after 24 h, and were elevated more significantly when in the combined therapy (Figure 5C). Taken together, the activation of the caspase-related apoptotic pathway may be one of the primary mechanisms by which TPL exerts its synergistic effect on CBP-treated A375 cells.

### 3.6 Combination of TPL and CBP was superior in inhibiting melanoma xenograft growth than either drug alone

**FIGURE 6:**
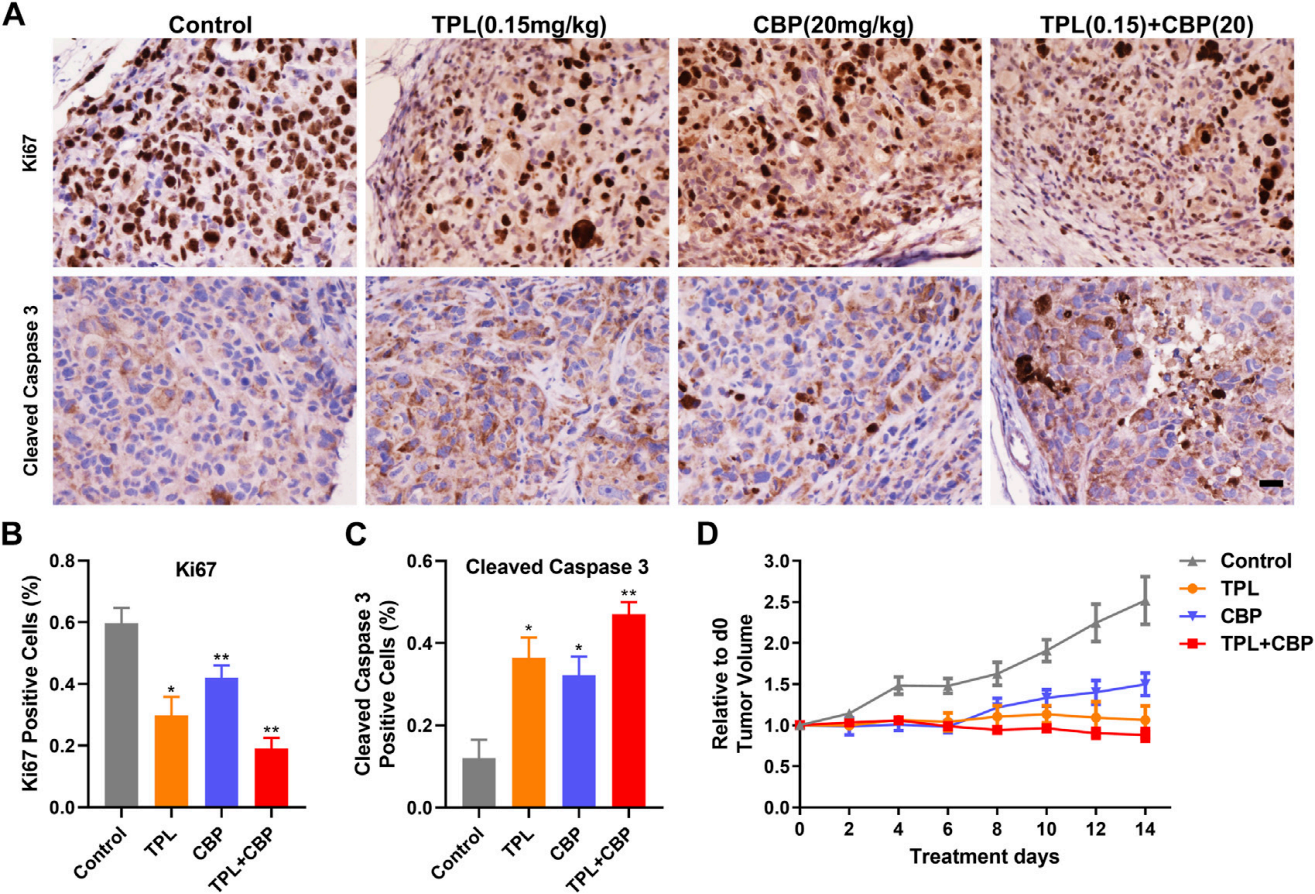
Effect of TPL and CBP on cell growth and apoptosis in a melanoma xenograft mouse model *in vivo*. **(A)** Representative images of Ki67 and cleaved caspase-3 stained tumor sections. Scale bar represents 50 μm. Immunohistochemical (IHC) analysis of proliferation and apoptosis in xenograft cells harvested from mice treated with Control (PBS), TPL (0.15 mg/kg), CBP (20 mg/kg) and Combined TPL (0.15 mg/kg) with CBP (20 mg/kg) by intravenously injection. **(B,C)** Quantitative analysis of Ki67 and cleaved caspase-3 stained IHC images randomly obtained from five mice. Mean ± SD values are presented. **(D)** Tumor growth curves of A375 cells in nude mice during the indicated treatment.

The antitumor effect of TPL and CBP was tested in a melanoma xenograft model. The A375 cells were transplanted into BALB/c nude mice for several days. The day of the Tumor volume (TV) reaching approximately 100-mm^3^ was counted as day 0. Then the tumor-bearing mice were randomized into four groups and injected intravenously with the indicated doses of drugs. Our results showed that TV was significantly reduced after treatment for 14 days. The growth of xenograft cells was inhibited when treated with TPL and CBP alone, but the therapeutic effect was more pronounced in combined treatment (Figure 6D). In addition, animals were weighed before and after treatment, and the change in body weight showed no possible serious adverse effects (data not shown).

IHC analysis of proliferation (Ki67) and apoptosis (Cleaved caspase-3) was determined in xenograft cells harvested from mice treated with indicated doses of drugs. Our results showed that tumor cell proliferation, as indicated by Ki67, was inhibited significantly by TPL or CBP treatment, especially in combined treatment (Figures 6A, B). In contrast, tumors treated with TPL combined with CBP had significantly increased cleaved caspase-3 expression, indicating a higher degree of apoptosis (Figures 6A, C).

### 3.7 TPL combined with CBP synergistically induces DNA damage by selectively inhibiting the NER activity in melanoma

**FIGURE 7:**
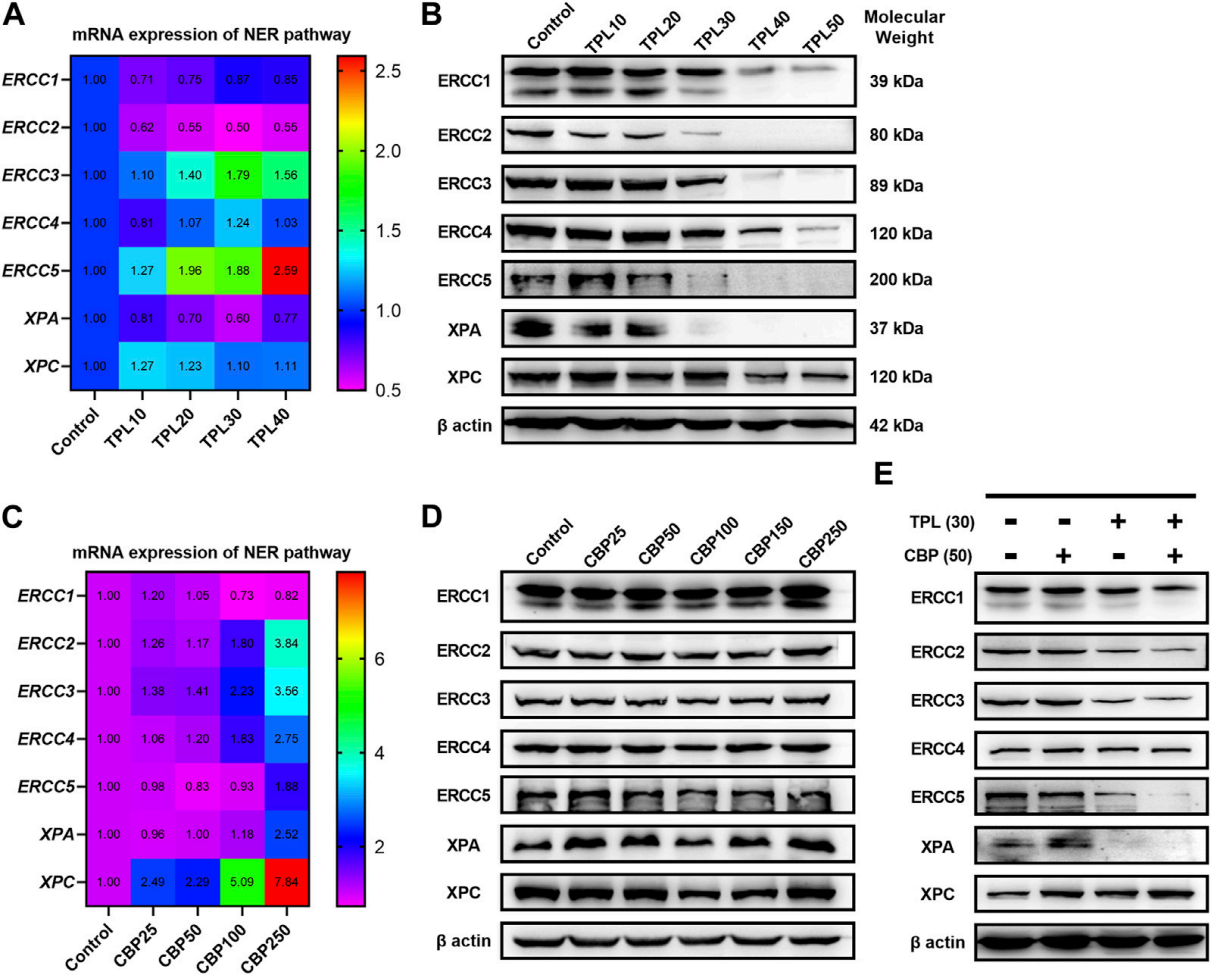
Effect of TPL and CBP on the mRNA and protein expressions of the NER pathway components. A375 cells were treated with TPL or CBP for 48 h with indicated concentrations. TPL abrogates the **(A)** mRNA and **(B)** protein expression levels of the rate-limiting components (ERCC1, ERCC2/XPD, ERCC3/XPB, ERCC4/XPF, ERCC5/XPG, XPA, and XPC) of the NER pathway, while CBP increases the **(C)** mRNA and **(D)** protein expression levels. **(E)** Effect of TPL (30 nM) and CBP (50 μM) alone or in combination on the levels of the NER pathway proteins in melanoma cells.

Since we had seen an increased extent of chromatin fragmentation in A375 cells treated with CBP and TPL, we wondered whether DNA repair capacity differed between TPL, CBP and co-treated groups. It is well known that DNA repair plays an important role in regulating the cytotoxicity of platinum drugs. NER is the major pathway of the cells to remove platinum-DNA adducts. This repair process mainly includes the recognition of the damaged DNA, the excision and removal of single-stranded damaged DNA fragments of approximately 30 base pairs, and the re-synthesis and ligation of the fragments to the pre-existing strand (Wang and Lippard, 2005). In this study, the mRNA and protein expressions of the molecules forming the repair complex were detected by PCR and Western blot assays. The rate-limiting proteins of the NER pathway (ERCC1, ERCC2/XPD, ERCC3/XPB, ERCC4/XPF, ERCC5/XPG, XPA, and XPC) were evaluated after treating A375 cells with TPL and CBP for 48 h. Our findings indicated that mRNA expression of the NER pathway genes in A375 cells increased after treatment with CBP in a dose-dependent manner (Figure 7C). On the contrary, the mRNA expression of *ERCC1*, *ERCC2,* and *XPA* decreased after treatment with TPL (Figure 7A). These results were further confirmed when we determined the protein expressions of these key components of the NER pathway (Figures 7B, D). However, the mRNA expression of *ERCC3*, *ERCC4*, *ERCC5,* and *XPC* did not change or increase after TPL treatment, indicating a direct effect of TPL at the protein level. Interestingly, the expression of these NER proteins showed an overall decrease when co-treated with 30 nM TPL and 50 μM CBP (Figure 7E). Therefore, TPL could abrogate the CBP-induced increase in the levels of the aforementioned NER pathway proteins.

**FIGURE 8:**
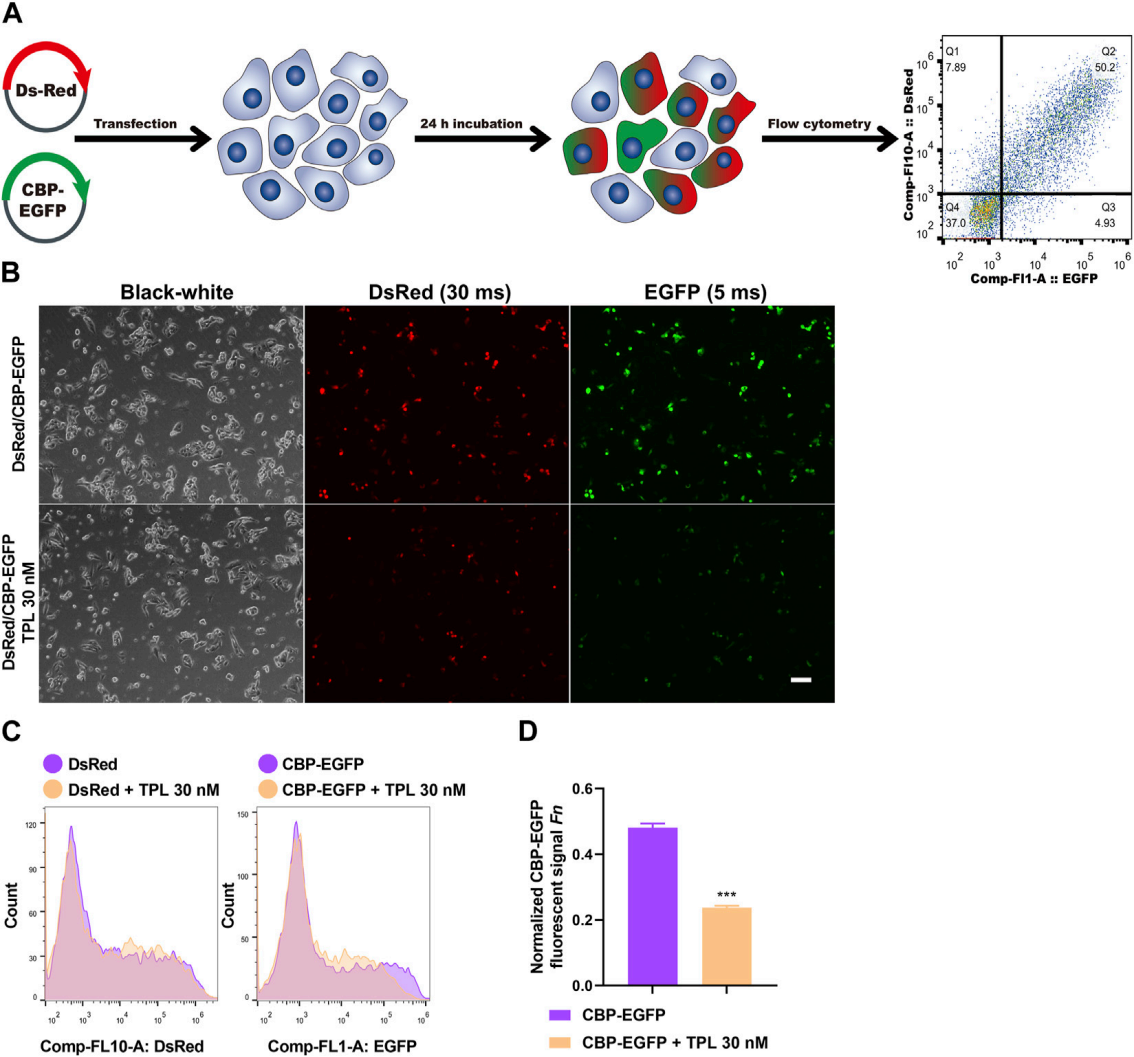
The effect of TPL on expression efficiency of CBP-damaged fluorescent reporter plasmids in A375 melanoma cells. **(A)** Assay workflow. Cells were transfected with undamaged DsRed fluorescent plasmid as a control for transfection efficiency, and co-transfected with CBP-damaged EGFP fluorescent plasmid (CBP of 50 μM, 37°C for 6 h) to determine the loss of fluorescent signal due to DNA damage between different groups. The transfected cells were cultured with or without TPL (30 nM) for 24 h. **(B)** Expressions of DsRed (30 m exposure time) and EGFP (5 m exposure time) fluorescent signals were simultaneously detected by a fluorescence microscope. The black-white images of the same view were also recorded to visualize the cells. Scale bar = 50 μm. **(C)** Cells were measured for fluorescence by flow cytometry. **(D)** Normalized EGFP fluorescent signal *Fn* was calculated to show the effect of TPL on NER repair capacity. Fluorescent signal was computed using the formula of “*F* = (*N* × *MFI*)/S". Where *S* is the total number of live cells and *N* is the number of EGFP or DsRed positive cells. *MFI* is the mean fluorescence intensity of the *N* cells. The DsRed fluorescence and EGFP signal was designated as *Fc* and *Fg*, respectively. The normalized EGFP fluorescent signal *Fn* was calculated using formula of “*Fn = Fg/Fc*".

To assess the effect of TPL on NER repair capacity, the CBP-damaged fluorescent reporter plasmid was used to determine the ability of the host cells to remove the platinum-DNA adducts that blocks transcription of the reporter gene in plasmid transiently transfected in A375 melanoma cells (Figure 8A). DNA lesions were introduced into the EGFP plasmid *in vitro* by incubating the plasmid with 50 μM CBP at 37°C for 6 h. Then the CBP damaged EGFP (CBP-EGFP) plasmid and the undamaged DsRed plasmid were combined together and co-transfected into A375 cells in the presence or absence of 30 nM TPL for 24 h. After incubation, fluorescent signals were observed by fluorescence microscope. In order to observe the fluorescence intensity expressed by different plasmids among different treatments, the exposure time for DsRed and EGFP was set at 30 and 5 m, respectively. Our results showed that the fluorescence intensity of DsRed decreased very slightly in the TPL treatment group, while the fluorescence intensity of CBP-EGFP decreased apparently (Figure 8B). The flow cytometry assay was used to measure the fluorescence intensity quantitatively. We found that TPL had little or very slight effect on the expression of undamaged DsRed plasmid. In contrast, TPL had a great effect on suppressing the expression of CBP-damaged EGFP plasmid in A375 cells (Figures 8C, D). The normalized CBP-EGFP fluorescent signal *Fn* decreased nearly two-fold after TPL treatment (Figure 8D). These results clearly demonstrated that TPL inhibits the CBP-induced DNA damage repair efficiency by the NER pathway.

## 4 Discussion

p53 is an important tumor-suppressor protein with critical roles in many cellular processes; especially when cells are stressed, cell proliferation can be inhibited by inducing cell cycle arrest or apoptosis (Geske et al., 2000; Benchimol, 2001; Oren, 2003). In addition, the p53 pathway can partially regulate CBP cytotoxicity, as p53 is also linked to DNA repair. Once DNA is damaged, p53 will be activated to trans-activate multiple downstream pathways, which in turn induces a variety of cellular responses. p53 pathway plays a core role in signal transduction, and also interacts with several key components of the NER pathway, such as XPC and TFIIH (Fan and Bertino, 1999), indicating that p53 also participates in DNA repair (Wang et al., 1995; McKay et al., 1999; Adimoolam and Ford, 2002; Adimoolam and Ford, 2003). In our current results, we found that co-treatment with TPL and CBP increased p53 expression. On the one hand, it can improve the repair capacity of DNA damage, and on the other hand, it can induce the unrepaired cells to apoptosis. However, proteins involved in p53 related pathways regulating p53 activity and CBP or TPL cytotoxicity had not been determined. Therefore, further studies of these related signaling molecules are still required to explore the specific role of p53 in the combination therapy of the two drugs.

**FIGURE 9:**
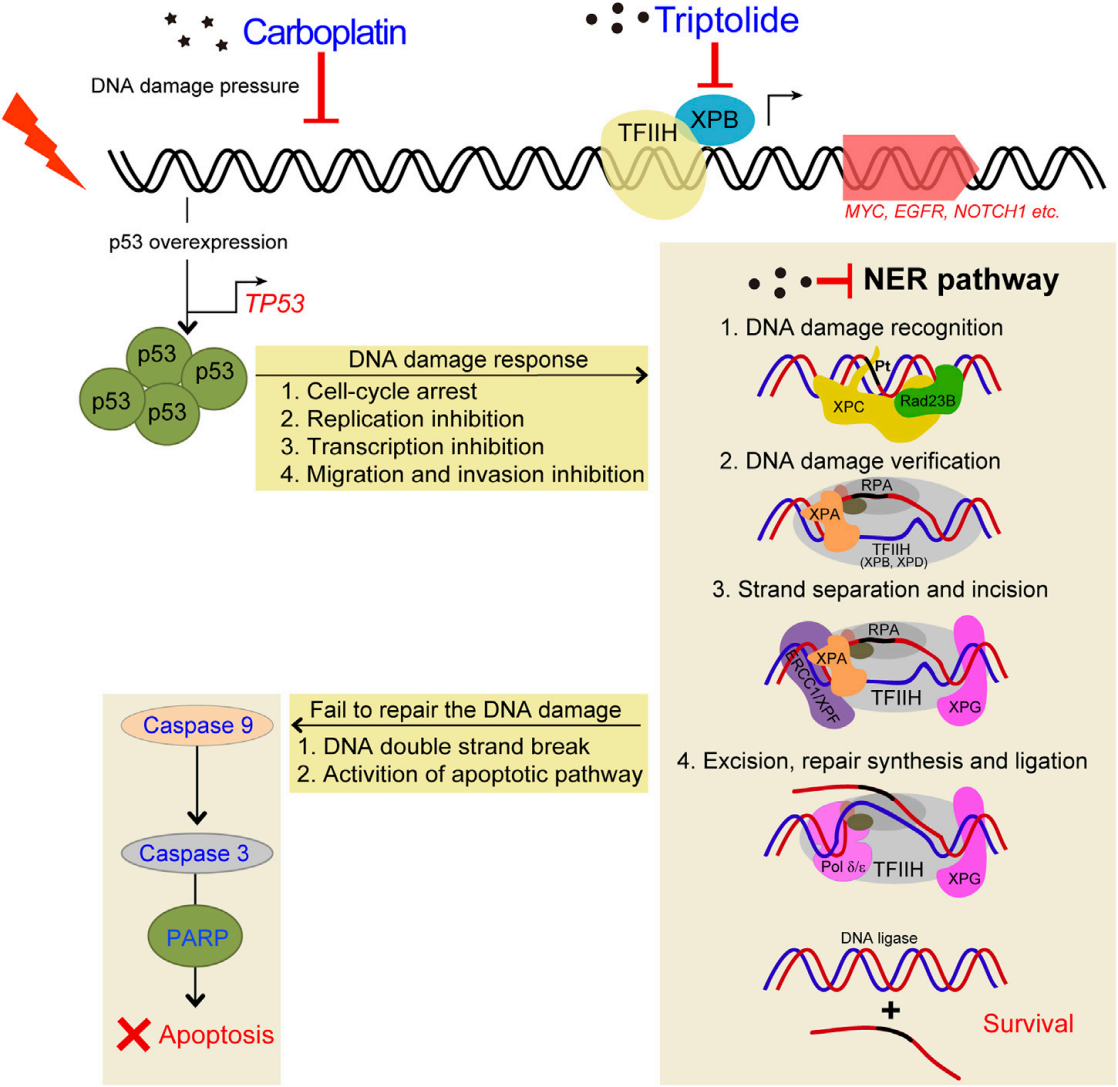
Schematic diagram demonstrating the inhibitory effects of TPL on CBP induced upregulation of NER pathway. TPL synergistically enhances CBP-induced apoptosis by selectively inhibiting the NER activity and overexpressing p53 in melanoma. The platinum induced DNA damage is identified and primarily repaired by the NER pathway. The damaged DNA can be recognized by XPC, and then XPA and RPA are recruited and act as another damage recognition complex that might participate in lesion verification. The DNA around the lesion site is opened asymmetrically in an ATP-dependent manner, employing the TFIIH complex with its two DNA helicases, ERCC3/XPB and ERCC2/XPD (Evans et al., 1997). TFIIH is involved in both transcription and NER processes. Subsequently, ERCC4/XPF-ERCC1 complex and ERCC5/XPG, two structure-specific endonucleases, incise on the 5′and 3′sides of the DNA adduct, respectively, to remove the damaged single-stranded DNA that contains the lesion. Finally, Pol δ/ε resynthesize the removed oligonucleotides and successfully repair the DNA damage by DNA ligase.

DNA repair plays an important role in regulating the cytotoxicity of platinum drugs. The efficacy of platinum-based chemotherapy depends on the DNA repair capacity of the treated tumor cells (Sarkaria et al., 2008). One of the most important mechanisms of resistance against platinum-based drugs such as cisplatin is the repair of drug-induced DNA damages through different signaling pathways, of which the most common is the NER pathway (Galluzzi et al., 2012; Abbotts et al., 2014). Indeed, tumor cells with increased NER capacity were often resistant to platinum-based drugs (Borst et al., 2008; Martin et al., 2008). NER is a major pathway of the damaged cells to remove platinum-DNA adducts (Figure 9). This repair process mainly includes the recognition of the damaged DNA, the excision and removal of single-stranded damaged DNA fragments of approximately 30 base pairs, and the re-synthesis and ligation of the repaired DNA fragments to the pre-existing strand (Costa et al., 2003). Furthermore, DNA double-strand breaks may also occur due to unrepaired DNA damage or after the removal of the intra-strand adducts. To adequately repair this damage, the homologous recombination is required (Borst, et al., 2008), which had not been determined in this study.

Cisplatin exerts its cytotoxicity by the covalent binding to purine bases of the DNA, which primarily produces inter-strand and intra-strand cross-linked DNA adducts (Kartalou and Essigmann, 2001). These DNA adducts can induce toxic DNA damage, mutations and rearrangement of DNA (Lawley and Phillips, 1996). The mechanisms of CBP and cisplatin are similar; both of them can generate DNA adducts, which will inhibit replication and induce cellular apoptosis if not an adequate repair machinery activity taken effect. In our study, TPL exhibits a synergistic effect with CBP in melanoma *in vitro* and *in vivo*. The mRNA and protein expressions of the NER components in A375 cells increased after treatment with CBP while they decreased after treatment with TPL in a dose-dependent manner. After treatment with CBP, the A375 cells were stressed by DNA damage and responded through overexpressing p53, which induced cell-cycle arrest, replication inhibition, transcription inhibition, migration and invasion inhibition. Then the NER pathway was activated by increasing the expression of the NER components. The damaged cells that were successfully repaired could continue to survive, and if not, the apoptotic pathway would activate, accompanied with DNA double-strand breaks (Figure 9). When combined treatment with TPL, the number of successfully repaired cells becomes less, due to the downregulation of NER protein expression by TPL. It has already been proved that TPL can bind to the TFIIH basal transcription factor ERCC3/XPB and decrease its expression (Titov, et al., 2011; He, et al., 2015). Recent studies reveal that TPL can interfere with various transcription factors, including p53 (Chang et al., 2001), nuclear factor kappa beta (NF-κB) (Qiu et al., 1999), heat shock factor protein 1 (HSF-1) (Westerheide et al., 2006), and nuclear factor of activated T-cells (NFAT) (Qiu, et al., 1999). In addition, TPL interacts with RNA polymerase II complex to induce a global suppression of RNA expression (Hou, et al., 2018), including the genes belonging to the NER pathway. Interestingly, our data showed that not all of the mRNA expression of the NER pathway genes decreased after TPL treatment. However, all of the protein expressions of these genes decreased. We speculated that in addition to binding to ERCC3/XPB, TPL might also bind to other NER pathway proteins, leading to the degradation of these proteins. On the other hand, TPL is considered to have multifunctional antitumor effects, and it can induce apoptosis via other pathways. Therefore, TPL has the potential to enhance the efficacy of melanoma cells for platinum-based therapy by disrupting the NER pathway and increasing apoptosis. In clinical, NER capacity measurements potentially might be used to personalize the DNA damage drug treatment modality in patients.

In our study, the NER repair capacity analysis was used to indirectly determine the ability of the host cells to remove the platinum-DNA adducts that blocks transcription of a fluorescent reporter gene in plasmid transiently transfected in A375 cells. Reporter gene expression could be reactivated by repairing transcriptional blockade lesions in the plasmid, thus providing a method to quantitatively measure the DNA repair capacity, which mainly through the NER pathway. Our fluorescent reporter plasmids studies further showed that TPL had little or very slight effect on the expression of undamaged DsRed plasmid. In contrast, TPL had a great effect on suppressing the expression of CBP-damaged EGFP plasmid in A375 cells (Figure 8). These results clearly demonstrated that TPL inhibits the CBP-induced DNA damage repair efficiency by the NER pathway. However, it is unclear whether other DNA repair-related pathways are also involved in the combined use of TPL and CBP. Nagel and colleagues used a method for measuring multiplexed DNA repair capacity (Nagel, et al., 2014). They have described an HCR assay using fluorescence-based multiplex flow cytometry that can identify different types of DNA damage in one test (Nagel, et al., 2014). In the future, we also need to explore the situation of other DNA repair pathways (base excision repair, mismatch repair, homologous recombination, and non-homologous end joining) after the combination treatment of the two drugs.

In conclusion, this study reveals that the presence of TPL in CBP treatment can selectively inhibit NER pathway activity, and TPL exerts a synergistic effect with CBP to induce apoptosis in melanoma. Based on our findings, TPL may be an inhibitor of the NER pathway that enhances platinum-based cancer therapy. The methods we used in this study provide a very efficient tool for studying the mechanisms of DNA damage repair and transcription, especially the NER pathway. Therefore, combined therapy with TPL and CBP might be an effective modality for the treatment of melanoma, which should be considered as part of a series of complementary analyses required for further evaluation in patients in the near future.

## Data Availability

The original contributions presented in the study are included in the article/Supplementary Material, further inquiries can be directed to the corresponding author.
