# Supplementary material for: Triptolide enhances carboplatin-induced apoptosis by inhibiting nucleotide excision repair (NER) activity in melanoma
Source: Front Pharmacol. 2023 Jun 1;14:1157433. doi: 10.3389/fphar.2023.1157433 (PMC10267402; doi:10.3389/fphar.2023.1157433)
Supplement: Supplementary file 1 [file Table1.DOCX]

**Table S1. The primers of nucleotide excision repair (NER) genes.**

| Official Symbol (Gene ID) | Official Full Name | Primer |
| --- | --- | --- |
| *ERCC1* (2067) | ERCC excision repair 1, endonuclease non-catalytic subunit | Forward: GACTGAATGTCTGACCACCG  Reverse: CTGGGGTCATCAGGGTACTT |
| *ERCC2/XPD* (2068) | ERCC excision repair 2, TFIIH core complex helicase subunit | Forward: CCTGGAGCAGCTAGAATCAG  Reverse: CTCTGGGTACCTGGTGGATA |
| *ERCC3/XPB* (2071) | ERCC excision repair 3, TFIIH core complex helicase subunit | Forward: GATGTTCCGAAGGGTGCTCA  Reverse: CTCCATCCAGTTGGCTTCGT |
| *ERCC4/XPF* (2072) | ERCC excision repair 4, endonuclease catalytic subunit | Forward: CTGGTCCTAGAAAGCAACCC  Reverse: CACCAAGAGCTTCACTCTCC |
| *ERCC5/XPG* (2073) | ERCC excision repair 5, endonuclease | Forward: ATATCTGGCTGTTTGGAGCG  Reverse: TTTCCATGGCGGTTACACAA |
| *XPA* (7507) | XPA, DNA damage recognition and repair factor | Forward: GTGTGGCCATGAACTGACA  Reverse: TGCACCTACTCTAGCACTCA |
| *XPC* (7508) | XPC complex subunit, DNA damage recognition and repair factor | Forward: GCCCATTTATGGACAGGGAG  Reverse: CTCCACGACAATACCCAAGG |
