# Supplementary figures and images for: Triptolide enhances carboplatin-induced apoptosis by inhibiting nucleotide excision repair (NER) activity in melanoma
Source: Front Pharmacol. 2023 Jun 1;14:1157433. doi: 10.3389/fphar.2023.1157433 (PMC10267402; doi:10.3389/fphar.2023.1157433)

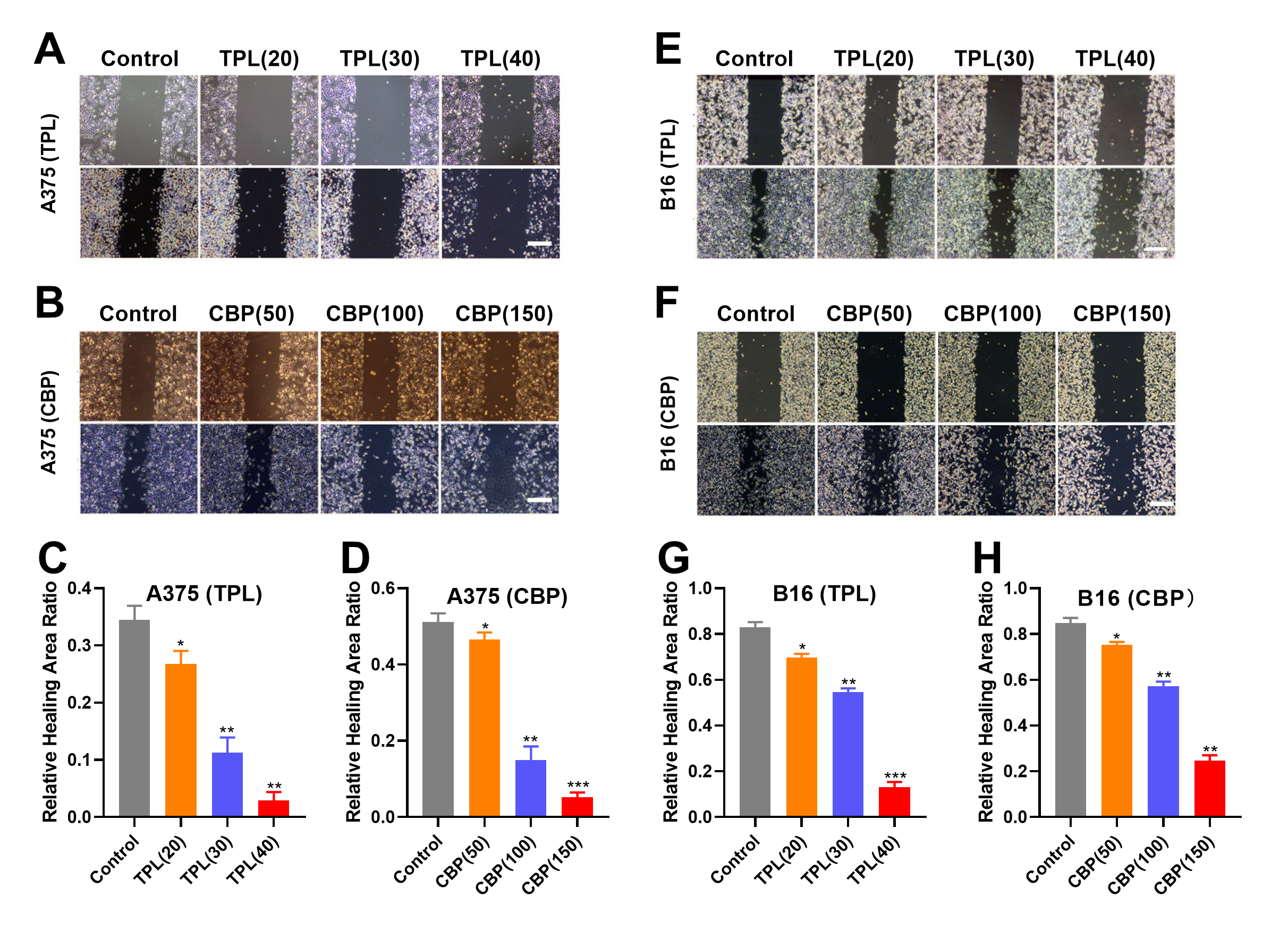

Supplement: Supplementary file 2 [file Image1.TIF]
