# Supplementary material for: Triptolide enhances carboplatin-induced apoptosis by inhibiting nucleotide excision repair (NER) activity in melanoma
Source: Front Pharmacol. 2023 Jun 1;14:1157433. doi: 10.3389/fphar.2023.1157433 (PMC10267402; doi:10.3389/fphar.2023.1157433)
Supplement: Supplementary file 3 [file DataSheet1.docx]

**Supplemental methods**

***In Vitro* Migration and Invasion Assays**

The wound-healing assay was used for the cell migration analysis. Briefly, the cells were grown to confluence in six-well plates, and a fixed-width wound in the cell monolayer was created with a sterile 10-μL micropipette tip. The cells were washed gently with PBS and treated with indicated concentrations of drugs, and incubated for 72 h. The subtraction of the “wound” area at the same site before and after treatment was calculated to determine the wound closure using ImageJ.

Cell invasion was measured by Matrigel-coated transwell cell culture chambers (8-μm pore size; Millipore, USA). Cells were cultured in serum-free medium for 24 h before treatment and were seeded in the upper chamber of the transwell insert with drugs for 48 h. However, the lower chamber contained a medium with 10% FBS. After 48 h, the invasive cells in the bottom of the chamber were fixed, stained, photographed and counted under a microscope.

**Immunofluorescence (IF) and immunohistochemistry (IHC)**

Cells were plated on collagen-coated coverslips in 24-well plates overnight, and treated with the indicated concentration of drugs for 48 h. Then the cells were fixed in 4% paraformaldehyde for 1 h, blocked in 2% goat serum, and incubated with indicated primary antibodies at 4°C in a wet box overnight. Alexa Fluor–conjugated secondary antibodies (Invitrogen) were added and incubated for one hour at room temperature. Coverslips were mounted in DAPI plus immunogold and imaged using an inverted fluorescence microscope (OLYMPUS IX71, Japan).

Formalin-fixed paraffin-embedded xenograft tumor sections were prepared according to conventional methods. After incubating the slides with primary antibodies, color development was performed using the HRP secondary antibody and DAB solution. IHC results were analyzed by Image-Pro Plus 6 software.

**Quantitative Real-Time RT-PCR**

A375 cells were seeded in 100-mm cell culture dishes overnight, and treated with TPL and CBP at indicated concentrations alone or in combination for 48 h. Total RNA from treated cells was isolated using Trizol reagent (Invitrogen, USA), and then reverse transcribed into cDNA for PCR assay using First-Strand cDNA Synthesis Kit (Promega, USA). SYBR Green Real-time PCR Master Mix (TOYOBO, Japan) was used to detect the mRNA expression on a LightCycler 480 detector (Roche Diagnostics Ltd, Switzerland). mRNA expression was normalized to the housekeeping gene *GAPDH* and analyzed using the 2^-ΔΔCt^ method. The primers of NER genes were synthesized by Sangon Biotech (Shanghai, China), and are listed in Table S1.

**Western Blot Analysis**

A375 cells were seeded and treated at indicated conditions. Total protein was extracted using RIPA buffer (Beyotime, China), and then added PMSF and phosphatase inhibitors (Beyotime) at 4°C.

After determining the protein concentrations by BCA (Beyotime) method, 30 μg protein from each sample was loaded into 8-12% SDS-PAGE to separate, and was transferred to a PVDF membrane using the wet transfer method. The membranes were blocked with 5% skimmed milk first and were then probed with indicated primary antibodies at 4°C overnight, followed by HRP-conjugated secondary antibodies. The enhanced chemiluminescence (ECL) kit (Beyotime) was used to visualize the immunoblots, as determined by densitometric analysis. The protein expressions were normalized to the housekeeping protein β actin.
